# Developing and Testing a User-Focused, Web GIS-Based Food Asset Map for an Under-Resourced Community in Northeastern Connecticut

**DOI:** 10.3390/nu17050911

**Published:** 2025-03-06

**Authors:** Xiran Chen, Manije Darooghegi Mofrad, Sydney Clements, Kate Killion, Thess Johnson, Xiang Chen, Donna Zigmont, Daniela C. Avelino, Brenda Lituma-Solis, Michael J. Puglisi, Valerie B. Duffy, Ock K. Chun

**Affiliations:** 1 Department of Nutritional Sciences, University of Connecticut, Storrs, CT 06269, USA; xiran.chen@uconn.edu (X.C.); manije.darooghegi_mofrad@uconn.edu (M.D.M.); michael.puglisi@uconn.edu (M.J.P.); 2 Department of Geography, Sustainability, Community and Urban Studies, University of Connecticut, Storrs, CT 06269, USA; sydney.clements@uconn.edu (S.C.); thess.johnson@uconn.edu (T.J.); peter.chen@uconn.edu (X.C.); 3 Department of Allied Health Sciences, University of Connecticut, Storrs, CT 06269, USA; katelizkillion@gmail.com (K.K.); donna.zigmont@uconn.edu (D.Z.); daniela_carolina.avelino@uconn.edu (D.C.A.); brenda.lituma_solis@uconn.edu (B.L.-S.); valerie.duffy@uconn.edu (V.B.D.)

**Keywords:** asset map, GIS, food security, digital food and nutrition literacy, low-income, usability test

## Abstract

**Background/Objectives:** Access to healthy and affordable food remains a challenge for under-resourced communities due to uneven food distribution and the need for reliable transportation. This study developed and evaluated an interactive Geographic Information System (GIS)-based food asset map for a low-income community in Windham, Connecticut to improve awareness of food resources and expand opportunities for fresh food access. **Methods:** Using the human-centered design (HCD) framework and the Asset-Based Community Development (ABCD) model, the map integrates food locations, transportation routes, and assistance eligibility. Internal pilot testing (n = 8) identified usability issues, leading to updates such as mobile compatibility and user guides. Usability testing (n = 74) assessed navigation performance and user feedback through task-based evaluations and surveys. Categorical map usability, sociodemographic, diet, and health characteristics were tested for participants with food security (yes/no) or digital literacy (passed/failed). **Results:** Food-secure participants showed higher usability success than food-insecure individuals (*p* < 0.05), while those relying on food assistance faced greater challenges (*p* < 0.05). Individuals rating their diet as “very good/excellent” were most likely to pass the map usability testing (*p* < 0.05), whereas younger, college-educated, employed participants and those with vehicles trended toward passing (*p* < 0.1). Participants generally reported the map easy to navigate, especially those with food security. **Conclusions:** The asset map promotes food resource awareness and addresses barriers such as limited public transportation information. Additional efforts are needed to support food-insecure users in utilizing digital food access resources. This study contributes to initiatives to improve food access, digital inclusion, and community engagement in under-resourced communities.

## 1. Introduction

Elevated risk of chronic diseases among under-resourced communities results from multiple social determinants of health that contribute to poor access to healthy, affordable, and culturally acceptable foods [1]. A nutrition-focused equity framework to promote health and prevent disease has been proposed that combines the socio-ecological levels (individual, interpersonal, community, policy) with multiple domains of influence (biological, behavioral, physical/built environment, socio-cultural environment, healthcare systems) [2]. The U.S. Department of Agriculture (USDA) has identified communities that are food deserts, characterized by high levels of poverty and low geographic access to supermarkets [3]. Food environments can also be classified as food swamps, where there is an overabundance of unhealthy food options such as convenience stores and fast-food restaurants, and limited access to fresh produce and healthy foods [4].

Research has shown that in food deserts and food swamps, low-income families face financial constraints and difficulty accessing healthy foods, resulting in poor dietary quality and increasing risks of chronic diseases such as obesity, diabetes, and cardiovascular conditions [5,6,7]. These environmental challenges directly contribute to food insecurity, defined as the difficulty individuals or households face in accessing adequate, healthy, and safe food due to limited economic resources [8]. However, food insecurity often extends beyond simple access, intersecting with nutritional insecurity, which focuses on deficiencies or imbalances in the nutrient content of available foods. Even when food is accessible, it may lack essential nutrients, such as vitamins and minerals, resulting in poor diet quality and adverse health outcomes [9].

The interplay between food insecurity and nutritional insecurity is particularly evident in food deserts and swamps, where the availability of nutrient-rich foods is limited, and residents are often forced to rely on inexpensive, calorie-dense but nutrient-poor options [10]. These intertwined issues highlight the complexity of addressing food environments in under-resourced communities, as they require not only improving geographic access to food but also ensuring the quality and nutritional adequacy of the available options. Addressing both dimensions is critical for improving overall health and reducing the risks of chronic diseases [11].

Attention to the food environment with different types of food resources and geographic locations is required not only to promote food security but also to provide residents with the ability to choose where to obtain their food, ultimately encouraging healthier food choices [12]. The food asset mapping concept, developed by experts in nutrition, public health, and geography, directly addresses these challenges by visualizing food resources within communities [13,14]. Asset maps provide a comprehensive overview of nearby resources, such as grocery stores, food pantries, and farmers’ markets, enabling residents to make informed decisions that align with cultural and dietary preferences. However, low-income communities often face uneven geographic distribution of food resources, with key resources concentrated in specific areas, forcing many residents to rely on less nutritious options from convenience stores or fast-food outlets [15]. Digital tools such as asset maps depend not only on geographic data but also on users’ digital skills and access to technology. Limited digital literacy, systemic barriers such as inconsistent internet connectivity, and non-intuitive map designs can all hinder the effective usage of these tools [16,17,18].

The needs assessment for the development of a GIS-enabled food asset map in this study draws on our recent qualitative interviews with community stakeholders in the target community, including the social services, transportation, and food retail sectors, to assess the key challenges faced by low-income residents in accessing healthy foods (manuscript under review). Key challenges included inadequate public transportation, rising food prices, limited knowledge of food resources available in the community, and a lack of culturally appropriate food supplies. These issues were exacerbated by the COVID-19 pandemic, which reduced transportation routes and increased reliance on convenience stores for low-nutrition, high-calorie foods [19]. Stakeholders noted that the termination of emergency Supplemental Nutrition Assistance Program (SNAP) benefits further strained household budgets, forcing many to prioritize less expensive processed foods over fresh produce. This trend highlights the importance of providing residents with tools that not only visualize available food resources but also empower them to choose healthier options within the constraints of their financial and transportation challenges.

The COVID-19 pandemic exacerbated food access challenges while simultaneously accelerating the adoption of digital tools [20]. Between 2019 and 2021, food insecurity among low-income households declined from 20.6% to 15.5%, largely due to emergency Supplemental Nutrition Assistance Program (SNAP) benefits and the expansion of online grocery shopping options [21]. However, the gradual elimination of emergency SNAP funding in some states by the end of 2022, coupled with an increase in the national food insecurity rate from 10.2% in 2021 to 12.8%, suggests that these improvements may have been temporary [21,22,23]. Additionally, rising food prices due to inflation have further strained household food budgets, increasing the risk of food insecurity [24,25]. The pandemic-driven shift toward digital food access solutions has demonstrated the potential of online grocery shopping and food resource mapping to enhance food security. However, many low-income households continue to face significant barriers to effectively utilizing these digital tools, including limited digital literacy, lack of awareness about available services, and difficulties navigating online platforms [17]. Addressing these challenges is crucial, as digital resources continue to play an increasingly important role in food access efforts, particularly for underserved populations [26].

A lack of awareness about food resource locations, eligibility for assistance programs, and online ordering options can significantly limit access to affordable, nutritious food, particularly in low-income, low-food-access communities. Research indicates that residents’ perceptions of the food environment influence food access behaviors, such as how proximity to supermarkets and fast-food outlets affects food choices and nutritional outcomes [13,27]. However, proximity alone does not determine access. Individuals must also be aware of where food resources are located, how to access them, and whether they qualify for assistance programs, while also facing potential digital barriers that hinder their use of online food access tools. Classifying food locations strictly as “healthy” or “unhealthy” can be biased, as most retailers offer a mix of food options, making it difficult to assess the true impact of geographic food availability on diet quality [28,29]. These challenges underscore the need for more comprehensive, real-time food access solutions that address both geographic awareness and digital accessibility barriers.

Geographic Information System (GIS) tools have been shown to be effective in improving food accessibility by mapping food resources, but their success depends on how well individuals can engage with them. Research indicates that digital literacy gaps, unfamiliarity with mapping interfaces, and barriers related to interpreting spatial information can limit the effectiveness of these tools [30,31]. Addressing the digital divide is essential to ensuring that GIS tools serve not just as informational resources but as practical tools for food acquisition. Prior studies suggest that simplified, user-friendly interface designs, structured digital literacy training, and integration with food assistance programs can enhance the usability and effectiveness of digital food access tools [32]. These strategies can help bridge the gap between awareness and action, ensuring that individuals not only know where food resources are located but can also effectively utilize available services.

To address these challenges, this study developed and evaluated an interactive web-based GIS food asset map designed to help low-income residents in Northeastern Connecticut (CT) identify and access food resources [33]. By providing real-time information on the location, hours of operation, and eligibility for food assistance programs (e.g., SNAP and the Special Supplemental Nutrition Program for Women, Infants, and Children (WIC)), including online ordering options, the map helps people gain greater access to healthier food options by allowing residents to select food resources based on their specific needs and preferences. Additionally, the study considered usability challenges that may arise due to varying levels of digital literacy, ensuring that the tool remains accessible and useful for a diverse range of users. Ongoing usability tests and community engagement will improve the map’s design and functionality, enhancing its effectiveness in enhancing food access for vulnerable populations.

## 2. Materials and Methods

### 2.1. Research Overview

This project developed a web GIS-based asset map of food resources specific to one low-income community in Northeastern CT. The map not only displays the operating hours and locations of these food resources but also empowers residents to choose where to obtain their food based on proximity, public transportation routes, and eligibility for food assistance programs. This emphasis on choice aims to encourage healthier food decisions and address key barriers to food access.

The geographic target location for this study is Windham County in CT, which faces numerous socioeconomic challenges that directly impact food accessibility and health outcomes. According to the ACCESS 2023 Community Needs Assessment, Windham County had the lowest per capita income and median household income in CT, with approximately 11.84% of residents living below the poverty line [34]. This included a high percentage of Hispanic/Latino populations, especially in Willimantic [34]. Furthermore, the 2021 American Community Survey (ACS) one-year estimates showed that the poverty rate in Northeastern CT was 11.6% [35], while Windham County’s median household income was the lowest in the state at USD 34,819 [36]. These economic conditions contribute to limited access to healthy, affordable, and culturally acceptable food options, exacerbating food insecurity and poor health outcomes. Windham County also reported the highest rates of diabetes (11%) and obesity (31%) in CT, both of which are closely linked to limited economic resources and insufficient access to nutritious foods [37]. Our team conducted a 2022 online survey of 276 adults in Windham County, where 17.4% of respondents reported food insecurity, a rate consistent with the expected food insecurity rate in CT during that period [38,39]. The COVID-19 pandemic significantly increased food insecurity rates across the state, especially for low-income individuals and families, with food access challenges intensifying in 2022 [38,40].

### 2.2. Theoretical Approach

The study used the human-centered design (HCD) framework and the healthcare access barriers (HCAB) model, along with the Asset-Based Community Development (ABCD) model, to guide the development of the GIS food map. The HCAB model identifies economic barriers (e.g., financial constraints), structural barriers (e.g., food swamps/deserts, limited public transportation), and cognitive barriers (e.g., inadequate digital skills and nutrition knowledge). These insights inform targeted intervention strategies [41,42]. The ABCD model, which focuses on leveraging community strengths and opportunities, ensures that residents actively contribute to identifying and utilizing available resources to drive development [43]. The HCD framework ensured that the map design reflected the needs and challenges faced by low-income, food-insecure communities. Community feedback informed decisions about design elements, including food resources (e.g., grocery stores, pantries, soup kitchens), navigation modes (e.g., driving, walking, public transit), and payment options (e.g., EBT, WIC). Combined with usability tests, this dynamic process ensured that the map evolved with user needs [44].

### 2.3. Asset Mapping

#### 2.3.1. Web GIS Development

The asset mapping process included data collection, geocoding, and visualization. Geodata were collected from public and commercial sources (e.g., food stores and public transit routes) and then cleaned and integrated into ESRI ArcGIS Pro. Food store locations were geocoded to generate a point layer, while Connecticut Department of Transportation public transit data were prepared in Shapefile format [45].

These datasets were uploaded to ArcGIS Online, and a web map was developed using Experience Builder. To ensure accessibility, three device-specific versions were created for desktop, tablet, and mobile to address the different digital resources available to community members. The map features an intuitive interface and search tool that allows users to filter resources based on specific criteria, such as EBT acceptance or public transportation options [30]. By integrating these filters, the map encourages residents to make food choices that are both practical and aligned with their dietary and cultural preferences. Community feedback is integral to the refinement of the map. Ongoing updates enhance search functions, simplify navigation, and ensure content accuracy. These improvements are designed to support low-income users with limited digital skills while maintaining a user-friendly experience [46].

#### 2.3.2. User-Focused Design

The map incorporates features tailored to the community’s food access needs, particularly those that rely on food assistance programs. It includes intuitive zooming, search tools, and clickable icons for grocery stores, pantries, and soup kitchens to ensure the map is easy to use for both community members and policymakers. To address the community’s reliance on assistance programs, filtering options allow users to locate food resources based on specific criteria:

Electronic Benefits Transfer (EBT) and Supplemental Nutrition Assistance Program (SNAP): Users can filter food retailers that accept EBT/SNAP, making it easier for low-income families to find participating businesses [47,48].
Women, Infants, and Children Special Supplemental Nutrition Program (WIC): This filter helps families find stores that offer WIC-eligible foods, ensuring that WIC recipients can easily access eligible items [48,49].
Online Grocery Ordering: The map includes a store filter that supports EBT/WIC online grocery shopping, which is convenient for users with mobility or safety issues. This feature is especially useful for families or individuals who are unable to visit stores in person [47,50].

In addition to food-specific filters and features, the map incorporates practical resource details to improve usability. Information such as store websites, detailed descriptions of food pantry/mobile pantry distribution sites, soup kitchen services, and contact methods (phone or email) are included. Navigation links, such as Google Maps integration, ensure users can easily locate resources. Additionally, public transportation details, including bus routes and stop locations, provide route-planning assistance, enhancing accessibility for individuals relying on transit.

### 2.4. Pilot Testing

Before deploying the food access map to the community, the research team conducted an internal pilot test to evaluate its functionality and user experience. The test aimed to identify usability challenges and collect feedback for improvement [51]. A convenience sampling method was used to recruit eight participants, including five research team members and three graduate students majoring in nutritional sciences. These individuals were chosen because they had not previously been involved in the development of the map to ensure unbiased feedback. Participants were informed of the purpose and procedures of the project and provided with a flyer with a QR code to access the map and accompanying survey. During the test, participants followed predefined usage scenarios, including searching for food stores, applying grocery store filters, and navigating to destinations. Staff provided initial guidance to ensure participants understood the process, but direct intervention was avoided during testing to preserve the integrity of the user feedback. We collected feedback on multiple aspects of the map, including interface design, ease of navigation, filtering capabilities, and overall usability. We systematically analyzed this feedback to identify potential areas for improvement. The testing process simulated real-world use cases to ensure that the results were relevant and actionable.

### 2.5. Usability Testing

#### 2.5.1. Participants

To evaluate the usability of a GIS-based interactive food map among low-income adults in Windham, CT, we recruited community residents into a single testing session between August and December 2024. The procedures were approved by the Institutional Review Board of the University of Connecticut. Participants signed an informed consent form, and their data were anonymized to ensure confidentiality. Eligible participants were adults living in Windham, CT, 19 years of age or older, and who speak and read English. Participants who completed the single testing session were given a USD 10 gift card.

#### 2.5.2. Procedure

The study team recruited participants in community settings that reach low-income adults with food or other resources. The single session took place in the community setting with participants using a personal electronic device (e.g., smartphone, tablet, or computer) to access the recruitment materials. To ensure eligibility, a study screener was accessible by QR code to confirm that participants were at least 19 years old and lived in the community. CAPTCHA verification prevented automated responses, and cookie technology ensured each participant submitted the response only once. Before proceeding, participants read an information sheet outlining the study and indicated their consent to participate by clicking to continue.

The usability test assessed participants’ ability to use the food access map to complete four specific tasks: finding the operating hours of a specific supermarket (“Can you use the food access map to check: When is Stop & Shop open on weekdays?”), identifying a mobile food pantry site on the map (“Can you find a mobile food pantry distribution site on the map?”), getting to the mobile pantry site from their home by navigating using Google Maps (“Can you use Google Maps to find how to get to the site of the mobile food pantry truck (walk, car, public transportation which you selected above)?”), and providing the address of a mobile pantry delivery location on the map (“What is the address of one of the mobile pantry distribution sites?”). For the first task, participants were presented with a multiple-choice question on operating hours and selected the correct answer from four options. For the second and third tasks, participants answered “yes” or “no” after using the map features. For the fourth task, participants typed the specific address of a mobile pantry site into a response field. The usability test was administered entirely via the Qualtrics platform, where all survey questions were designed, and participants submitted their responses. The test passing criterion was defined as all responses being correct. For yes/no questions, a “yes” answer was considered correct if the participant successfully used the map to complete the task. Participants who successfully completed all tasks without assistance were classified as passing the usability test. The usability test results were categorized as pass or fail based on this criterion.

Participants also completed a structured survey via the Qualtrics platform, which included socio-demographic characteristics, self-rated health and diet quality, food security status, digital resource access, and experience-based feedback on the usability and functionality of the food map. The food security assessment used the six-item USDA Food Security Survey Module [52]. Responses were scored on a scale from 0 to 6, classifying participants as food secure (0–1) or food insecure (2–6).

In addition to assessing food security, participants evaluated the Windham Food Access Map through specific usability and functionality questions in the Qualtrics survey. These questions were designed to measure the map’s ease of use and usefulness in locating food resources, utilizing five-point Likert scales. The first question assessed the ease of use, ranging from “very hard” to “very easy”, while the second gauged agreement with the statement, “This map helps me easily locate nearby sources of groceries and healthy food”, on a scale from “strongly disagree” to “strongly agree”. Responses for both questions were further categorized into levels of usability and perceived effectiveness to analyze the map’s performance in addressing participants’ needs.

#### 2.5.3. Data Analysis

Statistical analysis was performed using SPSS (version 28.0, SPSS Inc., Chicago, IL, USA). Descriptive analysis summarized the characteristics of the participants using frequencies and percentages. Chi-square tests were used to assess associations between usability test results and independent variables, including sociodemographic factors, food security, food assistance participation, and self-assessed health and diet status. Given the expected frequencies being less than 5 in some response categories in the assessment of perceived ease of use and usefulness of the food map, Fisher’s exact test was applied to compare differences between food-secure and food-insecure participants. Fisher’s exact test was chosen due to its suitability for small sample sizes, ensuring a more accurate estimation of statistical significance compared to the chi-square test [53]. Statistical significance was set at *p* < 0.05.

## 3. Results

### 3.1. Web-Based Asset Map

**Figure 1:**
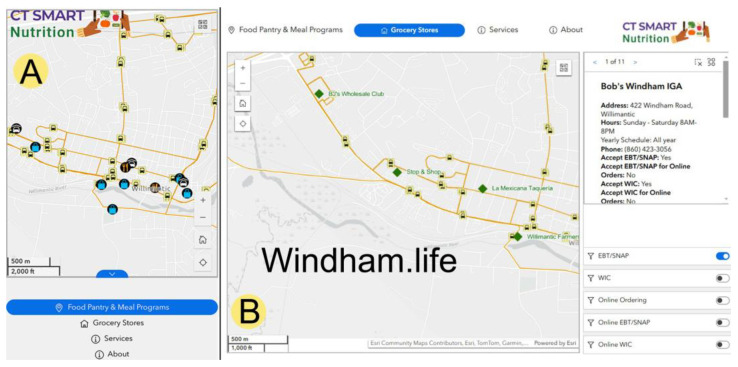
Windham Food Access Map (http://www.windham.life/ (accessed on 12 January 2025)) (**A**) mobile version displaying food assistance programs and (**B**) large-screen version displaying the grocery store page with store information and filters.

The Windham Food Access Map (http://www.windham.life/ (accessed on 12 January 2025)) tested for usability by community residents is an interactive, web-based asset map. The map integrates multiple layers of data, including the locations of food retailers, public transportation routes, filters to identify food retailers that accept different types of food assistance, and links to facilitate traveling to the food resource. Users can visually locate food resources on the community map with labeled roads, light gray and shaped building structures, and public bus routes in orange with clickable bus stop icons with sub-layers that show the bus route number. Figure 1A presents the mobile version of the map, reflecting the use of adaptive design principles for residents who may rely primarily on mobile devices, thus addressing one of the key findings from the community feedback regarding device accessibility.

These layers were combined to provide users with an interactive tool for visually locating food resources, including grocery stores, food pantries, mobile food distribution sites, and soup kitchens, each represented by distinct icons for easy identification, as shown in Figure 1B. These food resources had clickable and separate icon categories. Clicking the icon brought the user to a sub-layer showing the name of the agency hosting the food resource, the address, hours of operation, telephone number, additional notes affecting obtaining food, and a picture of the site. This feature ensures that residents can make well-informed decisions about where to obtain food and plan their visits effectively, addressing both informational and logistical barriers.

To further enhance user experiences, filtering options allow residents to identify food retailers that accept specific types of food assistance, including EBT/SNAP and WIC, as seen in Figure 1B. Additionally, a “How to get there” feature links directly to Google Maps, providing detailed route planning to reduce transportation barriers. This integration of transportation data into the food access map is one of the key outcomes of the study, designed to address the structural barriers to obtaining food that were identified in earlier research phases.

The visualization of these data layers, combined with intuitive zooming, panning, and clickable icons, allows users to quickly locate relevant food resources based on their individual needs. This interactive design ensures that even users with limited digital literacy can easily navigate the map and access important food resource information (see the pop-up sections in Figure 1B for details).

### 3.2. Pilot Testing Results

During the pilot testing process, the research team collected some feedback on the GIS interactive food asset map and made corresponding improvements based on this feedback as summarized in Table 1. First, some residents in the target community may prefer or need to access the map through a mobile phone. The team developed versions that are compatible with mobile phones and tablets. In addition, testers also reported that the small font size of the writing may be prohibitive for older adults and those with visual impairments. Therefore, the team added a zoom option to the map, allowing both a closer view as well as panning out to understand food resources relative to community landmarks. Some testers also pointed out that some residents of the target community may lack basic smart device operation skills and have difficulty using digital maps as well as linking to Google Maps. To address this issue, the research team will subsequently add simple and clear instructions on how to use the map to help residents familiarize themselves with the basic operating procedures. The map instruction is planned to include step-by-step visual instructions with clear screenshots and simplified text to accommodate different users. The nutrition education team also will provide hands-on training in the community. These efforts are aimed at ensuring that all residents, especially those with weak digital skills, can navigate the map effectively.

### 3.3. Usability Test Results

Our research team collected data from a total of 74 single sessions from residents in Windham, CT. Windham has the lowest median per capita income and median household income in CT [34]. Despite these challenges, the usability test results showed a higher rate of accessing the food map and answering the questionnaire using personal digital devices (such as smartphones, tablets, or computers) among all participants. This highlights the potential of leveraging technology to address barriers to food access for low-income populations. From the results of the participants’ map usability test, we also found that 24 of the 31 participants who failed the test answered “yes” regarding whether they could complete the navigation-related tasks, such as using Google Maps to find the location of mobile food pantries and determine routes to travel to them. These participants with a discrepancy between perceived navigating ability and demonstrated ability in the testing session also performed poorly in retrieving grocery store hours and accurately providing the address of the mobile food pantry site. This discrepancy suggests that while participants were able to navigate to food locations, they struggled with tasks requiring detailed information retrieval. This highlights the need for tools that better support decision-making and promote informed food choices, such as more intuitive design elements and guided assistance features.

Table 2 presents the demographic characteristics, food security status, self-rated health, and self-rated diet quality of participants who passed or failed the usability test of the food map. Young participants (aged 19–34) comprised the largest age group, representing 62.2% of the total sample. Although marginally significant (*p* = 0.091), the proportion of young participants was higher among those who passed the test than among those who failed. In contrast, participants aged 51 and older were more prevalent in the group who failed the test than in the group who passed. Marginally significant associations were found between usability test outcomes and participants’ education level, employment status, and number of drivable vehicles. Individuals with college or professional qualifications were more successful in the test, while those with only a high school diploma, technical training, or less (i.e., ≤8th grade/some secondary school) exhibited a higher failure rate (*p* = 0.064). Furthermore, participants who were employed (whether full-time, part-time, or self-employed) (*p* = 0.080) and those who owned at least one vehicle (*p* = 0.071) demonstrated marginally significantly higher pass rates than their counterparts.

Food security status and participation in food assistance programs emerged as significant predictors of food map usability. A significantly higher proportion of food-secure participants passed the test, while most food-insecure participants failed (*p* < 0.05). This suggests that individuals experiencing food insecurity may face additional barriers to acquiring or utilizing digital skills effectively, possibly due to limited access to technology or fewer opportunities for digital education. Similarly, a significantly higher proportion of participants who did not participate in food assistance programs passed the test, whereas those enrolled in one or more food assistance programs had a significantly higher failure rate (*p* < 0.05). These findings may reflect disparities in digital skills and access to technology, both of which can influence the effectiveness of digital tools aimed at improving food access.

Self-rated health status did not show a statistically significant relationship with usability outcomes (*p* = 0.325). In contrast, self-rated diet quality showed a significant association with food map usability (*p* < 0.05). Participants who rated their diet as very good or excellent were more likely to pass the test compared to those who failed, while those who considered their diet poor or fair had a higher failure rate than those who passed. These findings suggest that individuals with lower self-rated diet quality may face greater challenges in navigating and utilizing digital food resources effectively.

### 3.4. Associations Between Food Security and Map Usability

**Figure 2:**
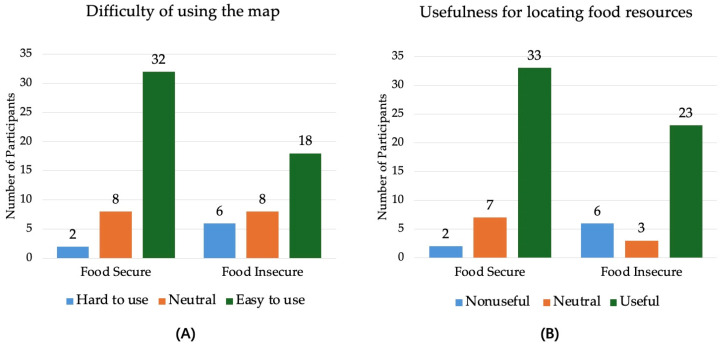
Feedback on map usage in terms of (**A**) difficulty in using the map and (**B**) usefulness for locating food resources. (**A**) Hard to Use includes option (Hard, Very Hard); Neutral includes option (Neutral); Easy to Use includes option (Easy, Very Easy); (**B**) Nonuseful includes options (Strongly Disagree, Disagree); Neutral includes options (Neither Agree nor Disagree); Useful includes options (Agree, Strongly Agree).

As shown in Figure 2, participants’ experiences with the Windham Food Access Map were evaluated in relation to their food security status. Two main usability outcomes were analyzed: (A) difficulty in using the map and (B) usefulness for locating food resources.

Figure 2 further analyzes the relationship between food security status and user experience with the Windham Food Access Map. As shown in Figure 2A, in terms of perceived difficulty, 32 of the 42 food-secure participants found the map “easy” or “very easy” to use, compared to 18 of the 32 food-insecure participants. Meanwhile, 6 of the 32 food-insecure participants found the map “difficult to use”, compared to only 2 of the 42 food-secure participants. Although this difference was non-significant (*p* = 0.101), the trend suggests that food-insecure individuals may need additional support to better use digital food access tools.

As shown in Figure 2B, in terms of perceived usefulness, 33 of the 42 food-secure participants agreed or strongly agreed that the map was helpful in finding food resources, compared to 23 of the 32 food-insecure participants; 6 of the 32 food-insecure participants found the tool useless, compared to only 2 of the 42 food-secure participants. Although this difference did not reach statistical significance (*p* = 0.143), the results highlight the importance of improving the usability of the map to ensure that the food-insecure population can more effectively access food resources through improved interface design and user training.

## 4. Discussion

The primary goal of the Windham GIS Food Map developed by the research team is to provide residents with an interactive virtual tool to find nearby healthy food resources, such as food pantries, grocery stores, and bus routes. By visualizing these resources, the map aims to address complex barriers to healthy food in low-income neighborhoods by leveraging technology to improve resource accessibility. The core purpose of such food maps is to help residents improve their awareness of food resources, create more opportunities for residents to access fresh food, and reduce disparities in food access. Through the use of GIS technology, maps can provide a detailed picture of a community’s food accessibility and reveal areas where ‘food deserts’, i.e., geographic spaces where residents have difficulty accessing healthy food, may exist [54]. However, while food maps show great potential in theory, there are still many challenges in practical applications. The usability test revealed that food-secure participants navigated the map more effectively than food-insecure participants, which may be attributed to their greater access to technology and digital literacy. Food-secure participants had a significantly higher map usability success rate compared to food-insecure participants, underscoring how food security is linked to better technological access and literacy. Additionally, participants in food assistance programs had lower usability success rates, suggesting the need for tailored design features, such as clearer navigation and simplified instructions, to better support this group. These findings demonstrate the important role that digital literacy and accessibility play in determining the effectiveness of GIS-based tools for low-income populations. Moreover, participants who rated their diet as very good or excellent achieved higher success rates on the map usability test. This suggests that a higher perceived diet quality may be linked to enhanced cognitive or navigational abilities, which in turn facilitate more effective use of digital mapping tools.

In addition to financial and technological barriers, transportation issues are also one of the main factors hindering access to healthy foods for low-income residents in a low-food-access community such as Windham County. A phenomenon described in the study by Wainer et al. is that many residents of low-income communities lack reliable transportation and are unable to regularly travel to distant supermarkets or farmer’s markets to purchase fresh food. Instead, they rely on convenience stores that are closer and cheaper. These convenience stores offer a limited variety of food and often sell processed foods that are less nutritious and more expensive, further limiting residents’ healthy eating options [55]. The usability tests in this study also revealed more insights into how transportation barriers and family composition affect food access. Furthermore, individuals who owned at least one vehicle performed better on the usability test. Although it was not statistically significant, we also found that participants in adult-only households tended to perform better compared to those in households with children. This disparity may reflect the significant time constraints and competing priorities faced by families with children, which can hinder their ability to navigate resources such as food maps or consistently plan trips to purchase healthy foods. Research from Ma et al. highlights that caregivers in food-insecure households with children often perceive limited access to affordable and high-quality nutritious foods despite geographical proximity to grocery stores. Additionally, minority communities are disproportionately affected by transportation barriers and the reliance on convenience stores with limited healthy options, exacerbating nutritional disparities [56]. These findings underscore the necessity of tailoring accessible food resources to the distinct needs of various households, emphasizing interventions that address perceived accessibility alongside geographic measures. Stakeholder interviews indicated that families in these communities purchase fresh vegetables and fruits less frequently due to transportation challenges, further exacerbating nutritional disparities [57]. Improving the supply of healthy foods in convenience stores could address this issue by increasing the availability of fresh, local, and affordable produce. This would allow residents to access more nutritious options even if they cannot travel far for groceries [58]. Enhancing public transportation services can significantly improve food accessibility, particularly for underserved minority communities. Sisk et al. pointed out that by optimizing the public transportation network and strengthening the connection between public transportation stations and food deserts, residents’ access to supermarkets and farmers’ markets can be effectively improved, thus reducing their reliance on convenience stores and improving the quality of their diet [59].

However, despite the GIS food map showing multiple food pantries that provide free or low-cost food to residents, the actual utilization of these resources by residents remains far below expectations. Through interviews, we found that many residents were not aware of the exact locations, hours of operation, or types of food provided by the pantries. Some even questioned the quality and freshness of the food and felt that it did not meet their cultural needs or dietary habits. Therefore, it is particularly important to raise the visibility of the pantries and ensure that the food they provide better meets the dietary needs of the residents. Partnering with nutrition education programs such as SNAP-Ed can provide residents with guidance to help them make healthier food choices, offer recipes that are culturally appropriate, or implement programs such as SWAP (Supporting Wellness at Pantries) to further empower residents [32].

Through a review of past projects, we found that existing food mapping projects typically focus on data collection and policy analysis to assess food access and community health. For example, Bader et al. examined how food mapping can help policymakers identify retail gaps [60], while Eckert et al. used GIS and demographic data to highlight communities with accessibility problems [61]. Hubley et al. mapped SNAP food stores and analyzed distances to assess their impact on accessibility [62], while MacDonald et al. used GIS to assess distance to sources of fresh food [63]. These studies provide valuable insights for policymakers but focus on macro-level policies rather than tools that directly improve daily food access and dietary quality of residents in need. Cohen et al.’s study shows that user-friendly food maps tailored to low-income communities can significantly improve access to healthy foods and improve nutritional status [32]. Unlike projects focused on policy and planning, our study addresses a real need by developing an accessible food map for Windham County. The map integrates community feedback and considers economic, transportation, nutrition, and digital literacy barriers. Future efforts should improve such tools to further improve food access and community health through real-time updates and technological advancements.

However, recent research on digital mapping techniques has also demonstrated the multiple values of GIS food maps. The Priority Food Index (PPFI) developed by Pontin et al. [64] highlights the effectiveness of GIS-based food maps in identifying and addressing food insecurity. They demonstrate how interactive GIS tools can support data-driven policy interventions by integrating geospatial and socioeconomic data to target food-insecure communities [64]. Unlike traditional static food maps, GIS-based food maps can dynamically display indicators of food resource availability and affordability, enabling decision-makers to design more effective interventions. Incorporating a similar approach into the Wyndham GIS food map could enhance its ability to provide real-time insights into disparities in food access and more effectively guide resource allocation.

Additionally, Mathenge et al. examined GIS-based spatial maps of food insecurity in Kenya and highlighted the importance of using multidimensional indices to assess barriers to food access beyond geographic proximity [65]. Their study found that GIS indicators combined with neighborhood analysis and socioeconomic data can reveal nuanced differences in food security across communities. This is consistent with our findings that food insecurity is determined not only by proximity to food retailers, but also by economic constraints, transportation barriers, and digital literacy gaps. The Wyndham GIS Food Map could be further improved by incorporating more granular indicators such as affordability metrics and user-reported food access challenges.

Another emerging area of GIS food mapping research focuses on real-time updates and improvements in digital accessibility. Mulrooney et al. discussed the role of real-time data collection and sensor integration in food mapping, highlighting how GIS technology can improve decision-making by constantly updating food availability information [66]. These findings suggest that future iterations of Wyndham GIS food maps should integrate dynamic data sources, such as real-time updates from pantries and grocery stores, to provide users with the most relevant and up-to-date information. Additionally, usability among food-insecure populations could be improved by enhancing the map interface to better accommodate users with varying levels of digital literacy, such as through the integration of step-by-step guides or voice-assisted navigation.

Furthermore, based on the results of the usability test, we can also find that digital literacy may not be a major barrier to participants using maps. According to Mocnik et al., the observed differences in usability tests may be more attributable to cognitive and representational barriers, such as difficulty understanding map symbols, spatial reasoning challenges, or cultural differences in interpreting geographic information [31]. Moreover, we found that participants with a college education achieved higher success rates on the map usability test, whereas those with lower levels of education were more likely to fail. Lack of formal education may lead to greater difficulty in understanding map symbols and navigational features. These findings suggest that differences in map-reading ability are not only unrelated to digital literacy but may also reflect deeper cognitive and experiential differences. Future improvements should therefore focus on designing more inclusive maps that provide more intuitive interfaces and step-by-step guides, while also strengthening digital skills training. Map usability could also be further enhanced by providing culturally relevant resources and nutrition guidance through partnerships with programs such as SNAP-Ed and SWAP (Supporting Wellness at Pantries) [32,67].

## 5. Strength and Limitations

A key strength of this study is its focus on community needs, emphasizing the practical utility of the map and incorporating community feedback to ensure it reflects the needs of the target population [68]. Additionally, the implementation of the GIS-based food map was designed to minimize costs, particularly by leveraging existing community resources and digital tools. The use of web-based GIS platforms reduces the need for extensive infrastructure investment, making it a relatively low-cost approach to improving food accessibility in low-income communities.

Despite its contributions, this study has several limitations. The small sample size restricts generalizability and limits the ability to analyze broader patterns of food access. However, the decision not to assess the association between the food map and dietary quality was not solely due to sample size constraints but rather a result of the study’s primary focus on the usability test. Additionally, as the study remains in the usability test phase, the long-term effectiveness of the food map in influencing food security and nutrition outcomes has not yet been evaluated. The research is also geographically limited to a single low-income community in Connecticut, which may not fully reflect the challenges faced by other communities with different food environments.

Moreover, the usability test revealed a discrepancy between participants’ self-perceived navigation abilities and their actual performance when using the food map. A considerable number of participants who believed they successfully completed navigation tasks struggled to access detailed information, such as identifying hours of operation or locating addresses. This suggests that some users may rely on assumptions rather than engaging with the map’s full functionality, highlighting the need for GIS tools that better support users with varying levels of digital proficiency. Future research should explore ways to enhance the interface design, provide clearer instructional guidance, and incorporate usability features that differentiate between basic and advanced digital navigation skills.

While the study evaluates usability barriers, it does not measure the extent to which digital literacy influences the ability to leverage GIS-based food mapping effectively. Future studies should address these gaps by expanding participant recruitment, incorporating diverse geographic regions, and conducting longitudinal evaluations. A more comprehensive assessment of food access improvements and their potential influence on dietary quality will be crucial in determining the long-term value of GIS-based food mapping for vulnerable populations.

Lastly, there are also financial constraints related to the maintenance and updating of food asset maps. While the web-based GIS approach helps reduce initial development costs, the use of commercial GIS tools, particularly the ESRI ArcGIS suite, does involve expenses for long-term maintenance, licensing, and data processing. These costs may pose challenges for food assistance programs, whose primary focus is providing quality food rather than sustaining digital tools. Thus, sustainable models for community-oriented GIS development, such as service-learning partnerships between universities and communities, leveraging open-source GIS (e.g., QGIS and Mapbox), and training local community members for long-term GIS maintenance, should be explored.

## 6. Conclusions and Future Research

This study demonstrates the potential of GIS-based food asset mapping in improving food access and resource visibility for low-income residents. While the current usability test phase provides insights into the practical application of the tool, further research is required to assess its long-term impact on dietary behaviors and food choices.

Future research should expand participant recruitment to include a more diverse population and conduct longitudinal studies to assess whether GIS-based food mapping translates into long-term dietary changes and improved food security. While this study provided initial usability insights, a larger and more representative sample is needed to understand variations in user experience across different demographic and socioeconomic groups. Further investigation should examine the role of digital literacy in map usability, evaluating how varying levels of digital skills influence food access behaviors and whether targeted digital literacy interventions could enhance the effectiveness of GIS-based tools in improving resource utilization.

Additionally, future studies should focus on optimizing the food map’s interface and functionality to better support diverse users. Enhancing navigation, improving accessibility features such as font size and contrast, and integrating multilingual support could make the tool more inclusive. Research should also explore how GIS-based food mapping can be integrated with community-based nutrition programs to address broader food access challenges. Expanding the study to neighboring communities and conducting longitudinal evaluations will provide a deeper understanding of the map’s role in improving food accessibility and dietary outcomes for low-income populations.

## Figures and Tables

**Table 1:** Summary of pilot testing: identified issues, description, and solutions.

Identified Issues	Description	Solutions
Mobile and tablet version	Residents may not have computers; mobile and tablet versions are needed	Developing mobile and tablet versions
Font size	Older adults with poor vision need larger font sizes	Adjusting font sizes
User guide	Some residents lack basic digital literacy, requiring a simple map usage guide	Developing a map user guide

**Table 2:** Sociodemographic, diet quality, and health characteristics of study participants in the usability test (n = 74).

	Map Usability Test ^7^		
Characteristic	% Participants (n) Who Passed (n = 43)	% Participants (n) Who Failed (n = 31)	*p* Value
Age			0.091
19–34 (n = 46)	72.1 (31)	48.4 (15)	
35–50 (n = 19)	20.9 (9)	32.3 (10)	
51+ (n = 9)	7.0 (3)	19.4 (6)	
Gender			0.943
Men (n = 29)	39.5 (17)	38.7 (12)	
Women (n = 45)	60.5 (26)	61.3 (19)	
Race and Ethnicity			0.976
White/Caucasian (n = 28)	39.5 (17)	35.5 (11)	
Latino/Hispanic (n = 19)	25.6 (11)	25.8 (8)	
Black/African American (n = 4)	4.7 (2)	6.5 (2)	
Others ^1^ (n = 23)	30.2 (13)	32.3 (10)	
Primary Language			0.557
English (n = 40)	51.2 (22)	58.1 (18)	
Non-English (n = 34)	48.8 (21)	41.9 (13)	
Household Type			0.177
Adults only (n = 56)	81.4 (35)	67.7 (21)	
Households with children (n = 18)	18.6 (8)	32.3 (10)	
Education			0.064
≤8th grade/Some High School (n = 5)	2.3 (1)	12.9 (4)	
H.S graduate/Technical (n = 28)	32.6 (14)	45.2 (14)	
College/Professional degree ^2^ (n = 41)	65.1 (28)	41.9 (13)	
Employment			0.080
Full-time/Part-time/Self-employed (n = 41)	60.5 (26)	48.4 (15)	
Unemployed, active seeking (n = 14)	11.6 (5)	29.0 (9)	
Unemployed, not seeking ^3^ (n = 13)	23.3 (10)	9.7 (3)	
Unable to work (due to disability or other reasons) (n = 6)	4.7 (2)	12.9 (4)	
Food Security Status ^4^			**<0.05**
Food secure (n = 42)	67.4 (29)	41.9 (13)	
Food insecure (n = 32)	32.6 (14)	58.1 (18)	
# of Drivable Vehicles ^5^			0.071
0 vehicles (n = 27)	27.9 (12)	48.4 (15)	
≥1 vehicles (n = 47)	72.1 (31)	51.6 (16)	
Food Assistance Program Participation ^6^			**<0.05**
None (n = 39)	62.8 (27)	38.7 (12)	
≥1 program (n = 35)	37.2 (16)	61.3 (19)	
Self-rated Health Status			0.325
Poor/Fair (n = 22)	23.3 (10)	38.7 (12)	
Good (n = 28)	39.5 (17)	35.5 (11)	
Very Good/Excellent (n = 24)	37.2 (16)	25.8 (8)	
Self-rated Diet Quality			**<0.05**
Poor/Fair (n = 29)	30.2 (13)	51.6 (16)	
Good (n = 29)	37.2 (16)	41.9 (13)	
Very Good/Excellent (n = 16)	32.6 (14)	6.5 (2)	

^1^ Includes American Indian, Asian, Native Hawaiian or Other Pacific Islander, and Puerto Rican. ^2^ Includes Bachelor’s degree, master’s degree, and higher. ^3^ Includes students, retirees, homemakers, disabled, etc. ^4^ Assessed using a 6-item short form of the Household Food Security Scale, scores range from 0–6, with 0–1 being food secure, 2–4 being Low food security, and 5–6 being Very low food security [52]. ^5^ Answer to the question “How many drivable motor vehicles (cars, trucks, and motorcycles) are there in your household?” ^6^ Includes Supplemental Nutrition assistance programs or food stamps (SNAP), the Special Supplemental Nutrition Program for Women, Infants, and Children (WIC), the Commodity Supplemental Food Program (CSFP), the Child and Adult Care Food Program (CACFP), the Farmers’ Market Nutrition Program (FMNP), the Senior Farmers’ Market Nutrition Program (SFMNP), the Temporary Emergency Food Assistance Program (TEFAP), food pantries, mobile food trucks, and food vouchers; ^7^ Usability test question included: 1. “Can you use the food access map to check: When is Stop & Shop open on weekdays?”; 2. Can you find a mobile food pantry distribution site on the map?; 3. Can you use Google Maps to find how to get to the site of the mobile food pantry truck (walk, car, public transportation which you selected above)? 4. What is the address of one of the mobile pantry distribution sites? The test passing criterion is POSITIVE answers to all questions.

## Data Availability

The raw data supporting the conclusions of this article will be made available by the authors on request due to restrictions containing information that could compromise the privacy of research participants.

## References

[1] Afshin A., Sur P.J., Fay K.A., Cornaby L., Ferrara G., Salama J.S., Mullany E.C., Abate K.H., Abbafati C., Abebe Z. (2019). Health effects of dietary risks in 195 countries, 1990–2017: A systematic analysis for the Global Burden of Disease Study 2017. Lancet.

[2] Agurs-Collins T., Alvidrez J., Ferreira S.E., Evans M., Gibbs K., Kowtha B., Pratt C., Reedy J., Shams-White M., Brown A.G. (2024). Perspective: Nutrition health disparities framework: A model to advance health equity. Adv. Nutr..

[3] United States Department of Agriculture Economic Research Service (2022). Food Access Research Atlas. https://www.ers.usda.gov/data-products/food-access-research-atlas/documentation/.

[4] Rose D., Bodor J.N., Swalm C.M., Rice J.C., Farley T.A., Hutchinson P.L. (2009). Deserts in New Orleans? Illustrations of Urban Food Access and Implications for Policy.

[5] DeWeese R., Ohri-Vachaspati P. (2017). Disparities in Healthy Food Access: Are We Improving?. FASEB J..

[6] Gopika G., Raghuveer V., MD V.K. (2022). A mini-review: Everything you need to know about food deserts. J. Environ. Sci. Public Health.

[7] Smets V., Vandevijvere S. (2022). The changing landscape of food deserts and swamps in Flanders, Belgium. Eur. J. Public Health.

[8] Definitions of Food Security (2023). Food Security in the U.S. https://www.ers.usda.gov/topics/food-nutrition-assistance/food-security-in-the-u-s/definitions-of-food-security/.

[9] Jones A.D., Ngure F.M., Pelto G., Young S.L. (2013). What are we assessing when we measure food security? A compendium and review of current metrics. Adv. Nutr..

[10] Olaboye J. (2024). Promoting healthy food access initiatives in urban areas of the USA: Strategies to address food insecurity and improve nutritional health. Int. J. Appl. Res. Soc. Sci..

[11] Ziso D., Chun O.K., Puglisi M.J. (2022). Increasing access to healthy foods through improving food environment: A review of mixed methods intervention studies with residents of low-income communities. Nutrients.

[12] Odoms-Young A., Brown A.G., Agurs-Collins T., Glanz K. (2023). Food insecurity, neighborhood food environment, and health disparities: State of the science, research gaps and opportunities. Am. J. Clin. Nutr..

[13] Li W., Cao S. (2024). Geographic poverty caused by distance to market. J. Infrastruct. Policy Dev..

[14] Luo Y., Ruggiano N., Bolt D., Witt J.-P., Anderson M., Gray J., Jiang Z. (2023). Community asset mapping in public health: A review of applications and approaches. Soc. Work Public Health.

[15] Romses K., Stephens T., Tran R., Crocker B., Lam V. (2017). Vancouver Food Asset Map helps users find food easily. Can. J. Diet. Pract. Res..

[16] de Jesus E.G.V., Brito P.L., de Oliveira Fernandes V. (2017). Interaction problems found through usability testing on interactive maps. Advances in Cartography and GIScience: Selections from the International Cartographic Conference 2017 28.

[17] Consavage Stanley K., Harrigan P.B., Serrano E.L., Kraak V.I. (2022). A systematic scoping review of the literacy literature to develop a digital food and nutrition literacy model for low-income adults to make healthy choices in the online food retail ecosystem to reduce obesity risk. Obes. Rev..

[18] Trude A.C., Lowery C.M., Ali S.H., Vedovato G.M. (2022). An equity-oriented systematic review of online grocery shopping among low-income populations: Implications for policy and research. Nutr. Rev..

[19] Cortés D.E., Zack R.M., Odayar V., Moyer M., Kumar A., Maia J.L., Bronico J.V.R., Granick J. (2024). The Impact of the COVID-19 Pandemic on Food Access: Insights from First-Person Accounts in a Safety-Net Health Care System. J. Health Care Poor Underserved.

[20] Woodward-Lopez G., Esaryk E., Rauzon S., Hewawitharana S.C., Thompson H.R., Cordon I., Whetstone L. (2023). Associations between Changes in Food Acquisition Behaviors, Dietary Intake, and Bodyweight during the COVID-19 Pandemic among Low-Income Parents in California. Nutrients.

[21] Troy A.L., Ahmad I., Zheng Z., Wadhera R.K. (2024). Food insecurity among low-income US adults during the COVID-19 pandemic. Ann. Intern. Med..

[22] Rabbitt M.P., Hales L.J., Burke M.P., Coleman-Jensen A. (2023). Household Food Security in the United States in 2022.

[23] Long C. (2023). SNAP Emergency Allotments Are Ending.

[24] Polsky J.Y. (2024). Trends in household food insecurity from the Canadian Community Health Survey, 2017 to 2022. Health Rep..

[25] Wells W., Jackson K., Leung C.W., Hamad R. (2024). Food Insufficiency Increased After the Expiration of COVID-19 Emergency Allotments for SNAP Benefits in 2023: Article examines food insufficiency after the expiration of COVID-19 emergency SNAP benefits. Health Aff..

[26] Evans A., Banks K., Jennings R., Nehme E., Nemec C., Sharma S., Hussaini A., Yaroch A. (2015). Increasing access to healthful foods: A qualitative study with residents of low-income communities. Int. J. Behav. Nutr. Phys. Act..

[27] Willis K.L. (2019). Beliefs and Opinions of Low-Income Residents Living in a Food Desert in a Gulf Coast State. Ph.D. Thesis.

[28] Glanz K., Sallis J.F., Saelens B.E., Frank L.D. (2007). Nutrition Environment Measures Survey in stores (NEMS-S): Development and evaluation. Am. J. Prev. Med..

[29] Chen X., Kwan M.-P. (2015). Contextual uncertainties, human mobility, and perceived food environment: The uncertain geographic context problem in food access research. Am. J. Public Health.

[30] Wamuyu P.K. (2017). Bridging the digital divide among low income urban communities. Leveraging use of Community Technology Centers. Telemat. Inform..

[31] Mocnik F.-B. (2023). Why we can read maps. Cartogr. Geogr. Inf. Sci..

[32] Cohen N., Ilieva R.T. (2021). Expanding the boundaries of food policy: The turn to equity in New York City. Food Policy.

[33] Soma T., Shulman T., Li B., Bulkan J., Curtis M. (2022). Food assets for whom? Community perspectives on food asset mapping in Canada. J. Urban. Int. Res. Placemaking Urban Sustain..

[34] Access Community Action Agency (2023). ACCESS 2023 Community Needs Assessment. https://accessagency.org/wp-content/uploads/2023/05/ACCESS-2023-Community-Needs-Assessment-7.pdf.

[35] United States Census Bureau 2021 ACS 1-Year Estimates Subject Table: Population for Whom Poverty Status is Determined-Percent Below Poverty Level. https://data.census.gov/map/050XX00US09110,09120,09130,09140,09150,09160,09170,09180,09190/ACSST1Y2023/S1701?layer=VT_2023_050_00_PY_D1&loc=41.5031,-72.8449,z8.3301.

[36] United States Census Bureau 2021 ACS 1-Year Estimates Subject Table: Median Income in the Past 12 Months. https://data.census.gov/map/050XX00US09001,09003,09005,09007,09009,09011,09013,09015/ACSDT1Y2021/B06011?layer=VT_2021_050_00_PY_D1&loc=41.5077,-72.8537,z8.1103.

[37] Data USA Health. Windham County, CT Profile. https://datausa.io/profile/geo/windham-county-ct#health.

[38] Avelino D.C., Duffy V.B., Puglisi M., Ray S., Lituma-Solis B., Nosal B.M., Madore M., Chun O.K. (2023). Can ordering groceries online support diet quality in adults who live in low food access and low-income environments?. Nutrients.

[39] Abraham M. (2022). DataHaven Survey Finds Food Insecurity Nearly Doubled in Connecticut in 2022. https://www.ctdatahaven.org/blog/datahaven-survey-finds-food-insecurity-nearly-doubled-connecticut-2022.

[40] Harper K., Belarmino E.H., Acciai F., Bertmann F., Ohri-Vachaspati P. (2022). Patterns of food assistance program participation, food insecurity, and pantry use among US households with children during the COVID-19 pandemic. Nutrients.

[41] Uansri S., Kunpeuk W., Julchoo S., Sinam P., Phaiyarom M., Suphanchaimat R. (2023). Perceived barriers of accessing healthcare among migrant workers in Thailand during the coronavirus disease 2019 (COVID-19) pandemic: A qualitative study. Int. J. Environ. Res. Public Health.

[42] Carrillo J.E., Carrillo V.A., Perez H.R., Salas-Lopez D., Natale-Pereira A., Byron A.T. (2011). Defining and targeting health care access barriers. J. Health Care Poor Underserved.

[43] Data Across Sectors for Health (2024). Asset-Based Community Development (ABCD). https://www.dashconnect.org/asset-based-community-development.

[44] National Institute of Standards and Technology Human Centered Design (HCD). Visualization and Usability Group 2021 May 3, 2021. https://www.nist.gov/itl/iad/visualization-and-usability-group/human-factors-human-centered-design.

[45] Mendez D.D., Duell J., Reiser S., Martin D., Gradeck R., Fabio A. (2014). A methodology for combining multiple commercial data sources to improve measurement of the food and alcohol environment: Applications of geographical information systems. Geospat. Health.

[46] Martin M., Peters B., Corbett J. (2012). Participatory Asset Mapping in the Lake Victoria Basin of Kenya. J. Urban Reg. Inf. Syst. Assoc..

[47] United States Department of Agriculture Food and Nutrition Service (2020). USDA Expands Access to Online Shopping in SNAP, Invests in Future WIC Opportunities. https://www.fns.usda.gov/news-item/fns-001820.

[48] WIC Research Policy Practice Online Shopping. https://thewichub.org/priority-issues/online-shopping/.

[49] SNAPtoHealth (2021). WIC EBT. https://www.snaptohealth.org/wic-2/wic-ebt/.

[50] United States Department of Agriculture Food and Nutrition Service Stores Accepting SNAP Online. https://www.fns.usda.gov/snap/online.

[51] Oulton K., Oldrieve N., Bayliss J., Jones V., Manning I., Shipway L., Gibson F. (2018). Using participatory and creative research methods to develop and pilot an informative game for preparing children for blood tests. Arts Health.

[52] Blumberg S.J., Bialostosky K., Hamilton W.L., Briefel R.R. (1999). The effectiveness of a short form of the Household Food Security Scale. Am. J. Public Health.

[53] Kim H.-Y. (2017). Statistical notes for clinical researchers: Chi-squared test and Fisher’s exact test. Restor. Dent. Endod..

[54] Walker R.E., Keane C.R., Burke J.G. (2010). Disparities and access to healthy food in the United States: A review of food deserts literature. Health Place.

[55] Wainer A., Robinson L., Argueta C.M., Cash S.B., Satin-Hernandez E., Chomitz V.R. (2023). At the nexus of grocery access and transportation: Assessing barriers and preferences for alternative approaches to enhancing food access. J. Transp. Health.

[56] Ma X., Liese A.D., Bell B.A., Martini L., Hibbert J., Draper C., Burke M.P., Jones S.J. (2016). Perceived and geographic food access and food security status among households with children. Public Health Nutr..

[57] Dubowitz T., Ghosh-Dastidar M., Cohen D.A., Beckman R., Steiner E.D., Hunter G.P., Flórez K.R., Huang C., Vaughan C.A., Sloan J.C. (2015). Diet and perceptions change with supermarket introduction in a food desert, but not because of supermarket use. Health Aff..

[58] Martin K.S., Havens E., Boyle K.E., Matthews G., Schilling E.A., Harel O., Ferris A.M. (2012). If you stock it, will they buy it? Healthy food availability and customer purchasing behaviour within corner stores in Hartford, CT, USA. Public Health Nutr..

[59] Sisk A., Rappazzo K., Luben T., Fefferman N. (2023). Connecting people to food: A network approach to alleviating food deserts. J. Transp. Health.

[60] Bader M.D., Purciel M., Yousefzadeh P., Neckerman K.M. (2010). Disparities in neighborhood food environments: Implications of measurement strategies. Econ. Geogr..

[61] Eckert J., Shetty S. (2011). Food systems, planning and quantifying access: Using GIS to plan for food retail. Appl. Geogr..

[62] Hubley T.A. (2011). Assessing the proximity of healthy food options and food deserts in a rural area in Maine. Appl. Geogr..

[63] Macdonald L., Ellaway A., Ball K., Macintyre S. (2011). Is proximity to a food retail store associated with diet and BMI in Glasgow, Scotland?. BMC Public Health.

[64] Pontin F., Baudains P., Ennis E., Morris M. (2023). Identifying drivers of food insecurity through linked data-the Priority Places for Food Index. Int. J. Popul. Data Sci..

[65] Mathenge M., Sonneveld B.G., Broerse J.E. (2023). Mapping the spatial dimension of food insecurity using GIS-based indicators: A case of Western Kenya. Food Secur..

[66] Mulrooney T., Wooten T. (2020). Digital high-scale food security analysis: Challenges, considerations and opportunities. International Conference on Geographical Information Systems Theory, Applications and Management.

[67] Lai J.S., Hiles S., Bisquera A., Hure A.J., McEvoy M., Attia J. (2014). A systematic review and meta-analysis of dietary patterns and depression in community-dwelling adults. Am. J. Clin. Nutr..

[68] Berggreen-Clausen A., Pha S.H., Alvesson H.M., Andersson A., Daivadanam M. (2022). Food environment interactions after migration: A scoping review on low- and middle-income country immigrants in high-income countries. Public Health Nutr..

